# Assessing the health impacts of implementing a ‘Comprehensive Rural Health Project’ health system in a low-income region of rural Nepal

**DOI:** 10.1371/journal.pgph.0004458

**Published:** 2025-04-29

**Authors:** Fred Barker, Radhakat Jha, Jasmine Morrish, Arbind Sah, Ramesh Choudhary, Richard W. Walker, Mike Lavender

**Affiliations:** 1 The Wolfson Institute of Population Health, Queen Mary University of London, London, United Kingdom; 2 Newcastle University, Newcastle-upon-Tyne, United Kingdom; 3 Northumbria Healthcare NHS Foundation Trust, Cramlington, United Kingdom; 4 Nepal Leprosy Trust, Lalgadh, Nepal; 5 Newcastle-upon-Tyne NHS Trust, Newcastle-upon-Tyne, United Kingdom; anusandhan trust (SATHI center), INDIA

## Abstract

Establishing and building grassroots, community-based healthcare systems is a key approach to improving healthcare access sustainably in low-income regions of the world. One prominent early example of this was the Comprehensive Rural Health Project (CRHP), inspiring the framework for subsequent large-scale programs globally. However, many community health projects do not provide the same breadth of services as CRHP, which may have impacts on health outcomes. This qualitative study focused on 12 Dalit villages in rural Nepal following an intervention - known as the Village Alive Project (VAP) - to boost healthcare provision through a CRHP-style health system. Villagers’ and health workers’ impressions of changes in healthcare access were assessed through 42 semi-structured interviews. Thematic analysis was performed using NVIVO by two independent authors; themes were finalized by reaching consensus. Three generated themes were shared by VAP and control villages: ‘changes in access to healthcare services’; ‘changes in health promotion and disease prevention’ and ‘inequalities and their effects on health’. A fourth theme, ‘views on the expansion of VAP to non-VAP villages’, was generated uniquely for the control group. Lack of health education and sanitation facilities, as well as social stigma, were listed as barriers to health prior to VAP’s establishment; most participants felt these have been largely addressed since the arrival of VAP. Implementing more comprehensive primary healthcare on top of pre-existing community-based healthcare systems is feasible, with encouraging findings from this low-income region of rural Nepal. Participants felt VAP improved understanding of diseases such as leprosy, which may benefit future vertical interventions. Improvements in various aspects of health and healthcare were reported for most or all study themes across intervention-group villages; improvements were also noted in control villages but with more evidence of ongoing barriers to health. Further studies looking at key quantitative outcomes are required to triangulate findings.

## Introduction

Universal healthcare access is listed as one of the United Nations’ Sustainable Development Goals to be achieved worldwide by 2030, owing to its significant focus towards global equity [1]. Strengthening primary healthcare systems in lower-income regions is a key strategy towards this goal [2], as it can address many of the most prominent health challenges while remaining holistic, accessible, cost-effective and sustainable [3–5]. One of the first and most notable examples of this being achieved was the Comprehensive Rural Health Project [6] (CRHP), focused on extremely socio-economically deprived Dalit caste villages around Jamkhed, India. CRHP trained locally sourced community health workers (CHWs) in each village to provide basic primary healthcare, who were supported by a single centralized hospital. The successes seen in Jamkhed have promoted the expansion of community workers globally [6–8], though the roles and enlisting requirements of these workers vary considerably across countries and regions [9–11].

In Nepal, CHWs have been described as ‘the backbone’ of the country’s healthcare system [12], contributing towards many of Nepal’s health improvements over recent decades [13,14]. However, issues with equity of healthcare service provision have been raised, particularly around the poorest regions of the country [15–17]. The Village Alive Project (VAP) was established in 2009 by the Nepal Leprosy Trust (NLT). It was set up in twelve extremely socioeconomically deprived villages across the Madhesh and Bagmati provinces in south-east Nepal, each with ongoing high rates of leprosy despite previous interventions by NLT. Anecdotal evidence suggested that vertical interventions used to tackle this challenge in the region were less effective in lower-income marginalized villages than they had been in higher-income villages. While community-based health systems and CHWs were present in Nepal by this time, they had been reported to have made little impact in such places, and villagers in the low-caste villages had reported difficulties accessing health centers on top of this.

VAP aimed to address these challenges, closely adhering to the original CRHP model. This system placed emphasis on training community health volunteers, known as Rural Health Facilitators (RHFs) to distinguish between themselves and state-funded CHWs. RHFs were each selected by their community based on several key factors: lived in the villages they served, were female (to be able to assist with childbirth in a culturally appropriate way); were at least semiliterate; were willing to leave their village to attend meetings; and were already a trusted member of the community. Training RHFs involved teaching in antenatal checks; assistance with childbirth; basic assessments; first aid and provision of health advice.

RHFs carry out a variety of roles, including but not limited to: establishing where facilities such as water pumps, toilets and washing areas were most needed and facilitating their construction accordingly (supported by funding from VAP); assisting with service provision for vaccination programs; dispensing supplements such as electrolyte replacement sachets, iron tablets, and vitamins; and establishing farmers’ and women’s groups to encourage sharing of experiences, skills and wisdom across the community. Aside from RHFs, VAP organized educational sessions to teach community members on health and sanitation, and their legal rights. These sessions were run either by local workers from government health facilities or by members of NLT staff. RHFs would receive small project payments to compensate them for the time spent on community health activities, though the majority of their day-to-day work was voluntary.

Unlike in the original CRHP model, VAP has not required construction of its own health clinics or hospitals due to the pre-existing provision of these as state public services. The second deviation between VAP and the CRHP is VAP’s establishment of financial savings groups for villagers. From this reserve, community members are able to take out small low-interest loans to fund local business ventures, or to provide short-term assistance in times of personal financial strain.

This study aimed to gain insights from villagers and RHFs regarding how the establishment of VAP had changed health and healthcare access in the area. Ongoing barriers to healthcare and the communities’ views towards their healthcare systems were also explored in detail. Findings were compared with those from two neighboring control villages.

## Methodology

### Ethics statement

Ethical approval was granted in the UK by the Faculty of Medical Sciences Ethics Committee at Newcastle University (ID 13898–2021), and in Nepal by the Nepal Health Research Council (ID 492/2021).

#### Interviews.

A total of 42 semi-structured interviews were carried out across 14 Dalit villages between 1^st^ November 2021–2^nd^ April 2022 across the Mahottari, Sarlahi and Sindhuli provinces of Nepal.

The inclusion criteria for this study required long-term residents of each village, who were at least eighteen years old and could speak either Nepali, a local regional language or English. Once the first participant was interviewed, we then aimed to interview a participant of the alternative gender for the second interview. However, this was not always possible due to most males in the villages working during the interview period. In cases where no males were available, a second female was interviewed.

14 villages in total were visited, in an order established by a random number generator. All 12 villages linked to VAP were included; in each case, the RHF and two further villagers were interviewed. The remaining 2 control villages were not associated with VAP, but were known to be similar geographically, demographically and socioeconomically; three participants were interviewed in these villages.

In cases where up-to-date maps of villages were available, locations to search for participants were established by dividing the map up into equal parts and using a random number generator to determine which part of the village to visit first. If no map was available, the village was subcategorized by someone familiar with the village, followed by using random number generation to determine where to visit. Once in this area, nearby local villagers were asked if they wished to take part in the study. If nobody was willing to do so, a new randomly selected area was visited next until somebody offered to participate. Interviews were carried out either in participants’ homes or in the village community centers. Participation was unpaid to minimize risk of bias.

Interviews took place in several regional languages (Maithili, Nepali and Thethi). All interviews were recorded using a SONY PX470 digital voice recorder, then uploaded onto an online database and transcribed by RJ into English within a few days of interviewing. Interview questions were similar for RHFs, VAP villagers and control villagers, adapted to fit each specific context; these are provided in (S1 Text, S2 Text, S3 Text). Semi-structured interviews adhered to relevant published guidance [18].

A sister study design was initially drafted alongside this study to assess pre-existing quantitative outcomes in VAP villages, with the intention of triangulating findings (further information can be provided through contacting the corresponding author). This sister study involved assessing pre-existing data from VAP on core health outcomes in these VAP villages. The quantitative study was not taken further and no quantitative analyses were carried out due to a lack of clear methodology available on how, when or by whom data were collected. Equally, no data were recorded from nearby control villages, so comparisons could not take place. The sister study included five multiple choice questions from participants, which were asked after completion of each interview. A table with all participant responses from this is provided in (S1 Table).

#### Data analysis.

All transcripts were coded by FB using NVIVO (Release 1.5.1). Inter-rater credibility checks were performed by an experienced external qualitative researcher (JM).

Codes were collated into themes for VAP and non-VAP villages separately by FB and JM independent of each other. Discrepancies in suggested themes were discussed until consensus was reached. Formal qualitative comparative analysis (QCA) was not carried out as part of this in this study.

## Results

Participant demographics are shown in Table 1. In total, 42 people took part in the study. All RHFs were female; 70% of the other participants were female. The median age of participants was 46.5 years. Most participants interviewed were Dalits who worked as farmers or laborers and were illiterate. The demographics of VAP villages were grossly similar to control villages, though there was a higher proportion of female participants in the VAP group.

**Table 1:** The table below shows the key demographics taken for each participant in our study, split according to whether they were part of the Village Alive Project villages or the control villages.

*Demographics*	VAP	Control
Total		
Gender	36	6
Male	6	3
Female (+ RHFs)	18 (+ 12)	3
Age		
30-39	9	1
40-49	9	3
50-59	6	2
60-69	12	0
Religion		
Hindu	36	6
Other	0	0
Caste category		
Dalit	26	5
Tribal	3	0
Other	7	1
Employment		
Farmer/laborer	24	6
Housewife	4	0
Tailor	3	0
Politics	3	0
Project coordinator	1	0
Merchant	1	0
Literacy		
Illiterate	27	5
Primary education	3	1
Secondary education	4	0
Adult literacy course	2	0

### Codes.

Codes from this study’s transcripts are available in (S2 Table, S3 Table).

### Themes.

In total, four themes were generated across VAP and non-VAP village interview subgroups; the first three were shared by both subgroups and focus on different aspects of health and healthcare access. The final theme focused on control villagers’ views on the possible establishment of VAP in their communities.

*Theme 1: Changes in access to healthcare services*

### VAP villages

Participants commented that the increased numbers of state health centers had improved healthcare access in the area. Participants felt RHFs played an important complementary role on top of this. RHFs could assess patients quickly and triage them appropriately to pharmacies, clinics or hospitals based on their presentations, helping the community to understand the complexities of the local healthcare system. RHFs and health promotion sessions encouraged patients with solely traditional health beliefs to increase their uptake of evidence-based medicine for important and treatable conditions. RHFs informed villagers about the presence of (and benefits of) available services such as vaccination programs, which the community had historically been reluctant to engage with.

“[Health in the community] was in a primitive stage [in the past]. If a person got sick, he was taken to a faith healer to find out whether a celestial soul was behind the sickness. Faith healers took longer to observe rituals and patients in the meantime got serious. This concept has changed now. They now know the cause of sickness and take the patient to a treatment center. Now we promptly send suspected leprosy cases to Lalgadh [the leprosy hospital] for confirmation. If confirmed, the treatment is started without any delay.”

-Male villager, Village 13

Participants felt that RHFs were well-trusted because they originated from the same village as themselves, thus having a personal stake in the health of their communities. RHFs were felt to be more accessible than other healthcare workers due to being available after 5pm if required. Their familiarity and accessibility improved community engagement with healthcare programs.

“Being the daughter in law of the village the community is fond of me. Therefore, I don’t hesitate to go to any one if my physical presence is more convenient to them.

-RHF, Village 12

Examples were provided where RHFs had positive impacts on maternal and child health, either through assisting with childbirth at home or triaging to secondary care at appropriate times.

“A lady in my village was in labor pain for two days. When this came to my knowledge, I went to that house and told the husband to take her to a birthing center immediately because you should only wait for 24 hours [in labor]. If the baby is not delivered in this time, it could be dangerous for both the mother and child. Therefore, she should be taken to a birthing center immediately.”

-RHF, Village 4

RHFs and VAP were able to provide up-to-date information to their communities during the coronavirus pandemic. RHFs have demonstrated an eagerness to continue learning about unfamiliar medical conditions and their management, suggesting they are willing to expand their breadth of knowledge in the face of new challenges.

“The inception of VAP assured us of a better future. During the coronavirus panic we were alerted to keep social distancing, keep off from strangers, and to request the municipality to make shelter for those who were suspected Coronavirus carriers.”

-Female villager, Village 5

“We are ready to accept any additional responsibility that is given to us.”

-RHF, Village 12

Some ongoing barriers to health were raised by participants. These were generally related to a lack of trust in state funded or private healthcare providers, either due to complaints of poor quality service or from suspicion of the over-medicalization of minor health issues. However, there were suggestions that villagers felt doctors took their presentations more seriously when an RHF attended the hospital with them.

“Whenever we go for treatment [at the health centers], they don’t let us come closer. They talk from a distance and throw medicines at us as if we were coronavirus patients. (…) The government hospital is worthless for us. They have neither medicines nor the staff.”

-Female villager, Village 7.

### Control villages

Participants feel health access has improved following the arrival of local health centers and hospitals. Participants noted that villagers have a lower threshold for seeking medical help than they did in the past, and often do this shortly after becoming unwell. Many villagers now indicate a preference for seeing doctors first, with further input from traditional healers afterwards if required. However, some participants still demonstrated an ongoing preference for traditional healers.

“[Healthcare access] is easier now. In the past health facilities were fewer. One had to travel long distances to receive treatment. Nowadays, almost all villages have some kind of health facility where one can get treatment conveniently.”

-Male villager, Village 8

Similarly to in VAP villages, concerns were raised that doctors and other healthcare staff are not always available at publicly funded centers, requiring villagers to pay for expensive private clinics. Participants also expressed doubt as to whether private workers were truly invested in the wellbeing of their communities.

“Government doctors are also involved in private practice. If we can’t find them in the hospital, we have to go to their private clinics. (…) Influential community people get all the privileges from the hospital already, so they don’t mind what the doctors do to poor and illiterate people.”

-Male villager, Village 8

“[Doctors] have a vested interest to do private practice. As a result, they are reluctant about promoting positive changes and educating people for better health.”

-Male villager, Village 9.

*Theme 2: Changes in health promotion and disease prevention*

### VAP villages

Participants felt their communities significantly benefitted from teachings from weekly education sessions provided by VAP (and reiterated by RHFs in the communities) on a broad range of topics relating to health. There is evidence that these sessions have led to long-term changes towards healthier lifestyles.

“When we started working with the Village Alive Program, the staff came every week and conducted talk programs on topics of health, sanitation and cleanliness of the village. Only then we realized the importance of these components. Following initiation from VAP staff we conducted a cleanliness program and our village looked better than ever.”

-Female villager, Village 12

“[Expecting mothers] didn’t tend to agree to take iron pills and injections. They now know what to do during pregnancy. In the past, three or four malnourished children had been detected, but in the last three years no malnourished child has been detected”

-RHF, Village 5

While not the only organization to do so, participants commented positively on VAP’s provision of water pumps, toilets and washing facilities to each village. Positive consequences of this included minimizing the necessity for open defecation, ensuring plentiful safe drinking water and allowing people to clean themselves in a safe environment.

“[In the past] there was an acute shortage of drinking water. When VAP came to the village, four hand pumps were installed through the initiation of the groups and by the courtesy of VAP, thus the problem of drinking water was solved.”

-Female villager, Village 14

However, one participant expressed concerns over the allocation of water sources, which he felt was unfairly distributed to those close to the RHF rather than equally across the community.

“Lalgadh sent some set of tube wells to be distributed among the members. Some of them formed another committee and bored tube wells as per their wishes without the consent of the committee. (…) Some of them are bored at their kith and kin’s place.”

-Male villager, Village 4

Participants raised some ongoing public health concerns they felt were not currently being fully addressed by either VAP or government posts. These were predominantly related to environmental factors such as the negative effects of pesticides, ultra-processed foods and alcoholism. Participants also commented on noticing a rise in the frequencies of non-communicable diseases such as cardiovascular disease and cancer.

“The only thing remaining to be abolished from the village, and that is the excessive use of alcohol in the village. They’re the ones who create nuisance in the family and community.”

-Female villager, Village 7

“People get sicker now because of the excessive use of synthetic fertilizers and insecticides and fungicides in the crops which we use as our food. Besides, many processed foods are in the market which tempts the children.”

-Female villager, Village 10.

### Control villages

Control participants also noted general improvements in village cleanliness. Practices such as open defecation have reduced largely due to external state pressure. There is good evidence of progress in the provision of toilets and other basic sanitation through support from organizations such as the United Nations Development Program, Red Cross and NLT.

“The municipality has made it mandatory for the villagers to construct their own toilet. We have been forced to make our own toilet or otherwise the municipality will not give any support to villagers who disobey. And so, people were forced to make toilets. Now they are happy about this decision. Fewer people now get sick.”

-Male villager, Village 8

However, facilities were reported to be considerably more variable in quality and have been poorly maintained in many cases. Typhoid, a disease transmitted through the fecal-oral route, was mentioned as an ongoing issue in one of the two control villages. There was little evidence of understanding about the origins of disease, particularly communicable diseases.

“Our village has only two tube wells. One tube well is not functioning, so only one is working for this population. (…) Everyone uses it, but no one keeps it clean. They don’t know their responsibilities. What can you do?”

-Female villager, Village 9

“Many toilets are not in use due to lack of maintenance and some people do not have enough land to construct one. Those caught defecating outside in the open have to pay a fine of 500 rupees. Open defecation has reduced but landless people have no option - they continue to go in the open.”

-Male villager, Village 9.

*Theme 3: Inequalities and their effects on health*

### VAP villages

VAP has been felt to place most focus towards the poorest people in each village. Most participants felt this represented a fair allocation of resources.

“Poor and deprived people benefit the most. (…) They are short of resources, so they need help on a priority basis.”

-Female villager, Village 3

Many participants felt that VAP establishing and running women’s and farmers’ groups has helped to encourage cooperation within communities, allowing these historically marginalized groups of villagers to feel more confident and empowered. This has allowed them to address local challenges that they would not otherwise have felt able to do.

“We have learnt about our rights after joining this group. I am not an educated person. I learnt things only after coming in contact with VAP.”

-Male villager, Village 2

Participants noted large improvements in the degree of stigma and discrimination seen in villages following the arrival of VAP. This included stigma for many diseases such as leprosy, (which until recently was believed to be secondary to a curse), as well as discrimination related to gender and caste.

“[VAP] has helped in reducing stigma to a great extent. Before VAP was implemented here, there was severe discrimination in the community. Nobody let us sit closer to them nor would they let us eat from the same plate. But once VAP started operating here, health education was given to those affected [with leprosy] as well as to the community people, and it had a very good outcome. During training we came to know that once you start taking treatment, leprosy is no longer communicable. We have since slept on the same beds together.”

-Male villager (with leprosy), Village 3

Despite efforts from VAP, some ongoing issues were raised. Firstly, villagers noted that factors such as the coronavirus pandemic had made group meetings more infrequent compared with in the past. Some participants felt there were persisting issues of inequality in their villages; a prominent example was an RHF’s experience of verbal abuse against her by her partner after realizing her influence on improving women’s status in the community:

“There is male supremacy over the women. My husband doesn’t let me speak and says that if he had known the consequences of giving me the freedom of advocacy for women, he would never have let me do it. We are chided by males for being more vocal. They accuse us of being more logical and persuasive. ‘Did I give you the opportunity to be a leader in the community to see you in this state? Do the sirs of VAP teach you these things? I will thrash you if you go any further’ roars my husband.”

-RHF, Village 4.

### Control villages

There were limited data from participants regarding discrimination in their villages, though many did not feel it was a pressing issue. However, one participant, in spite of denying that discrimination was present in her community, expressed a hesitancy to eat food prepared by Dalit families.

“We would not eat [a Dalit’s] food but if our children eat at their place, we would not mind. Since we worship our family deity every day, we don’t want to eat at their place.”

-Female villager, Village 8.

*Theme 4: Views on the expansion of VAP from control villages*

Participants expressed general interest in the program, and many offered their services to work with VAP if it was to expand to their villages. Participants felt that the program would help with community building and helping people know which health posts to go to.

“[VAP] could be a very good thing for the community. If I am asked for help, I would not leave even a single stone unturned for the betterment of the program.”

-Male villager, Village 8.

## Discussion

A major finding of this study is that it demonstrates that the implementation of a CRHP-like system on top of pre-existing low-income healthcare systems is feasible, and has been executed successfully in this region. The overall impressions of villagers is that the arrival of VAP has had significant positive impacts on their villages overall.

RHFs live in (and often grew up in) the villages that they work, and are therefore familiar with local values and customs. Being known and trusted in their villages was listed as a key factor that helped RHFs to engage and mobilize their communities for positive change. Such engagement is already known to be important in order to improve health through community-based healthcare interventions [19]. In this particular context, Dalits have experienced a long history of oppression from others in society. In situations where similar work was carried out by an external worker, marginalized communities may be less likely to engage fully or as quickly due to concerns as to whether they could be trusted or not. Furthermore, research exists suggesting locally sourced (rather than external) healthcare workers may have better long-term retention rates, ensuring more sustainable healthcare access [20]. A trusted local RHF therefore acts to bridge the gap between these historically marginalized communities and their health system.

RHFs demonstrated a willingness and capacity to adapt to evolving health challenges in our study. This study was carried out shortly after the COVID pandemic, where RHFs had a role in encouraging the use of face masks and keeping space between people from different households. This adaptability will be increasingly important as populations age, as frailty and non-communicable diseases will bring significant new challenges to many low-income countries’ healthcare systems [21].

The importance of health education has been well studied, and is known to be beneficial when carried out to a high standard [22]. The provision of educational sessions in VAP villages challenged preconceived ideas about disease that in many cases prevented patients from seeking medical care. For example, a person discovering they have leprosy may feel the need to hide their symptoms, believing this to be an untreatable curse and fearing social stigma if this were discovered by the community [23]. In comparison, the awareness that leprosy is a treatable bacterial condition encourages prompt treatment, preventing the condition from becoming disabling and lowering stigma in turn. Our findings show that metaphysical health beliefs are still prevalent in these communities, though general understandings of disease and health are now far more in line with evidence-based medicine since VAP intervention.

The creation and oversight of women’s and farmers’ groups in VAP villages were positively received by participants, reflecting an essential rebalancing of power back to those who have been particularly socially and economically vulnerable. Encouraging women to take leadership roles as RHFs helps to create positive female role models for villagers, working towards challenging gender inequality in communities where issues are evidently ongoing [24].

The authors note that many public health improvements in the region have been supported through a commendable increase in healthcare facilities, funded through state investment. While community programs are important to improving health in rural regions, they will always require healthcare posts to run effectively to ensure patients who need urgent medical care can access it.

### Comparisons *between* intervention and control villages

Formal qualitative comparative analysis (QCA) was felt not to be beneficial to this study for various reasons. Firstly, the nature of our study was explorative and responses were unpredictable, complex and heavily contextual to each village. It was therefore hard to establish relevant scoring variables for meaningful and reliable comparisons prior to the interview stage. Secondly, the study prioritized visiting all VAP villages (to get a full breadth of participants’ experiences of the intervention) instead of achieving a balanced number of VAP and control villages. This low number of control villages visited would therefore make QCA results more unreliable and make confident conclusions difficult. Instead, more general comparisons are made in this section.

Compared with participants from control villages, those from VAP villages generally described stronger, more valuable relationships between their community and their healthcare systems. Many villagers in VAP villages noted that they would go to an RHF if they had any health issues who would try to help them, and if unable to will refer them to the appropriate health center. In comparison, most respondents from control villages noted that they would likely to directly to a hospital or health center if they had a health issue. This may suggest that the local CHW is not being consulted on a regular basis. However, this is not certain from our data, as it is not clear where these workers are specifically based for each village.

Another key difference was that RHFs were available to help at any point in the day compared to the standard working hours of a CHW. This accessibility brings both benefits and possible issues - in cases where a villager needs help out-of-hours, such as going into labor at home overnight, support from an RHF would be invaluable but would not be possible by a CHW. However, RHFs may become vulnerable to burnout if they are too frequently called upon at unpredictable or unsocial hours. Burnout is known to negatively impact job retention, satisfaction and performance [25,26]; it is therefore important that steps are in place so that workers can discuss any issues and they can be addressed appropriately.

Another major difference was the effect of educational sessions, which were only available in VAP villages. In many cases, VAP villagers demonstrated a good understanding on the reasons given behind why certain health behaviors have been encouraged since VAP’s arrival. In comparison, the control groups show less clear evidence of this, and in many cases changes in behaviors are largely driven by financial threats from government as opposed to an understanding of the reasons why such changes are recommended. There is evidence that understanding the logic behind the advice that is given is likely to have more significant and longer-term improvements in behavior change over simply providing instruction [27,28], supporting VAP’s approach. Furthermore, once the knowledge is present within a community it can theoretically be continuously taught from villager to villager thereafter, continuing the benefits of education onwards.

Water pumps and toilets have been provided both to VAP and control villages, though in greater quantities to VAP villages. The greater quantity of facilities has led to more positive experiences and more hygienic habits than those reported in control villages. The report of both a malfunctioning and poorly maintained water pump in one of the control villages reiterates problems that arise without health advocacy; such jobs could theoretically be addressed by a role such as the RHF.

It is difficult to establish a reliable quantification of levels of discrimination and stigma from our study alone. Participants from VAP villages reported significant improvements in stigma locally, particularly for Dalits, women and those with conditions such as leprosy. The control participants we interviewed denied significant inequalities in their villages, though we had limited data on how they felt this had changed over time. Our findings still show evidence of ongoing inequalities in both control and VAP villages, so more research would be required to investigate this more closely before firm comparisons can be made.

## Strengths and limitations

There were several strengths to this study. A large number of interviews were carried out and all VAP intervention villages were visited. This enabled gathering of a broad range of perspectives from individuals and communities and ensured key events in all villages were included in analysis. The study actively explored both developments, and ongoing barriers, to health within this population and acknowledged factors such as social wellbeing within its analysis. Comparisons with control villages gave insight into developments that were unique to VAP, and highlighted differences in local challenges between VAP villages and controls. Our paper focuses primarily on a comprehensive CRHP-style system, which place additional emphasis on education and community mobilization. While these factors are common aspects of community health worker programs, there are no universal criteria for which roles and duties are required of community health workers. This leads to significant variation in the design of programs and various levels of success for particular programs [29,30]. Further research would be needed to explore this relationship in more detail to establish which factors are most important to the success of community-based health programs.

The study had a number of limitations. Only two control villages could be visited due to funding restrictions. This reduced the chance that saturation could be reached from control data, and was a factor partially precluding QCA. As a result, the comparisons that could be made were more modest than what may have been possible from a fully comparative study. Due to COVID travel restrictions delaying the interview phase of this study, most interviews could only be carried out during the regional harvest season, which limited the number of working participants available to take part in the study. Additionally, no included villagers worked abroad for employment, though this makes up a notable part of the local male population; missing their perspectives may have limited our range of findings. Finally, the study’s translator and co-interviewer (RJ) was a retired employee of VAP, which may have led to conscious or unconscious bias during the interview and translation processes. This highlights a local research challenge, as we were unable to find an available co-interviewer whom was able to speak English and the necessary regional languages, as well as read and write, but whom had no link to VAP. The authors aimed to mitigate risk of bias in other ways as much as possible by: utilizing randomness to aid participant selection in villages; explaining to participants prior to commencing that interviews are for the purposes of health research rather than for granting money locally; drafting questions specifically exploring issues and barriers to healthcare; and drafting questions to ask for specific examples of participants’ witnessed experiences of what has changed, rather than simply asking their opinions on topics.

## Conclusion

This study has demonstrated evidence that a CRHP-style health system in a considerably socioeconomically deprived region of rural Nepal is viable and is well-received by the villages it serves. CRHP is already a validated health intervention in Jamkhed, India [31,32]; this study demonstrates that the system brings similar social and health benefits to similar areas elsewhere. This study highlights mechanisms in which the ‘horizontal’ healthcare system acts synergistically with other ‘vertical’ approaches to target diseases such as leprosy in the area. To the authors’ knowledge, this is the first paper that compares two distinct variations of community-based health interventions in Nepal.

Our results suggest that similar low-income areas that are falling behind on health outcomes may benefit from expanding their service provision towards a CRHP model. Noting shared characteristics between CRHP and VAP villages, areas with the following characteristics may be particularly likely to benefit from increasing to more comprehensive community health systems:

- Populations who are seldom accessing primary or secondary care services when necessary, as a result of financial, social or logistical factors.
- Populations lacking an evidence-based understanding of disease and health (to the significant detriment of their health).
- Areas lacking the capacity to prevent common but preventable diseases.
- Areas heavily affected by stigma relating to class, gender or disease.

In order to gain further insight into the true value of CRHP-style interventions both in Nepal and elsewhere, high-quality quantitative analyses of key outcomes against controls and cost-benefit analyses will be required in future studies.

## Supporting information

S1 Text This section represents the list of planned questions asked to VAP villagers in our qualitative study, including prompts to ask for further examples in some cases. (DOCX)

S2 Text This section represents the list of planned questions asked to Rural Health Facilitators in our qualitative study, including prompts to ask for further examples in some cases. (DOCX)

S3 Text List of planned questions asked to VAP villagers in our qualitative study, including prompts to ask for further examples in some cases. (DOCX)

S1 Table Results from a brief questionnaire of all included participants. This questionnaire was part of a separate quantitative study which was not carried further primary due to unreliable overall data quality. Some issues with the quality of the responses were noted by FJB upon observing the transcribed data. Unfortunately, the interviewer RJ was not aware of the requirement to always request a specific response from the participant. In such cases, the given answer had to be inferred by FB from the overall interview (marked in the table with a ‘?’). Secondly, certain responses such as ‘health center’ or ‘hospital’ were ambiguous, as they appeared to be used interchangeably by participants in interviews during out our qualitative paper. No further analyses were carried out on these findings as a result; they have been included here for the sake of transparency.
(DOCX)

S2 Table List of initial transcript codes from across VAP villages. The middle row reflects the number of transcript files (interviews) with this particular code. The right row is the number of times the code was coded overall.
(DOCX)

S3 Table List of initial transcript codes from across control villages. The middle column reflects the number of transcript files (interviews) with this particular code. The right column is the number of times the code was coded overall.
(DOCX)
