# Supplementary material for: Assessing the health impacts of implementing a ‘Comprehensive Rural Health Project’ health system in a low-income region of rural Nepal
Source: PLOS Glob Public Health. 2025 Apr 29;5(4):e0004458. doi: 10.1371/journal.pgph.0004458 (PMC12040125; doi:10.1371/journal.pgph.0004458)
Supplement: S1 Text — (DOCX) [file pgph.0004458.s001.docx]

**S1 Text - Supplementary information 1**

[Legend] This section represents the list of planned questions asked to VAP villagers in our qualitative study, including prompts to ask for further examples in some cases.

Section 1: demographic information

Age:

Gender:

Ethnicity:

Caste:

Education level:

Job:

Section 2: Semi-structured interviews

1) This line represents your life from the point you were born (on the left) to now (the right). Please comment on the major changes (improvements or otherwise) to the health of **your village** that have taken place over the course of your life. Please explain why these measures were beneficial.

Today

Your birth

2) How would you describe the health of your village and local area at present?

3) Who do you and your family see for your health needs? What kind of services/treatments do you see them for and why?

4) What have been your general impressions of the Village Alive Project and the Rural Health Facilitators (please highlight that it VAP is separate from the government community program)?

- - Why?

5) What are your impressions on the difference in roles between the government community health program and the VAP Rural Health Facilitators?

- Do you believe the two workers complement each other’s work?
- Do you have any examples of this?

6) In your view, has healthcare access changed (improved or otherwise) because of the Village Alive Project?

- If positive:
  - Can you give any examples?
  - Of these, which interventions have been the most significant and why?
- If no change/access is worse:
  - Why do you think this is?
  - Has any intervention in particular made this worse?

7) Who do you feel benefits the most from the Village Alive Project?

- Why is that?
- Do you think it is fair/important that these people get most benefit?

8) Who do you feel benefits the least from the Village Alive Project?

- Why is that?
- Do you think it is fair that these people get least benefit?

9) Have there been any developments in your understanding of your own health and the ways to improve it since the introduction of the Village Alive Project (please again specify that this is separate from the government program)?

- - Can you give any examples?
  - Do you feel any more could be done by the Village Alive Project to help you learn more about your own health?

10) Has anything made it difficult for you to access healthcare since the implementation of the Village Alive Project and the Rural Health Facilitators?

- Cultural factors?
- Social factors?
- Financial factors?
- Logistical factors?

11) How much trust do you have in the ability of the Rural Health Facilitators to be able to help you or your family when you need them?

- Why?
- Has anything happened in particular to influence your opinion?
- Is there anything that you think could improve this?

12) Are there any additional roles that you feel the Rural Health Facilitators could be doing to improve the health of the area?

- Can you give more information into why you think this would be beneficial?

13) What are the biggest health challenges that the area still currently faces?

- Do you think VAP has the ability to address these challenges?
  - If so, why and how?
  - If not, why not?

14) Are there any other local challenges that you feel might be preventing the area from improving its own health?

- - If so, why and how?
  - Can you see a way that the Village Alive Project could help?
