# Supplementary material for: Assessing the health impacts of implementing a ‘Comprehensive Rural Health Project’ health system in a low-income region of rural Nepal
Source: PLOS Glob Public Health. 2025 Apr 29;5(4):e0004458. doi: 10.1371/journal.pgph.0004458 (PMC12040125; doi:10.1371/journal.pgph.0004458)
Supplement: S3 Text — (DOCX) [file pgph.0004458.s003.docx]

**S3 Text - Supplementary information 3**

[Legend] This section represents the list of planned questions asked to VAP villagers in our qualitative study, including prompts to ask for further examples in some cases.

Section 1: Demographic information

Age:

Gender:

Ethnicity:

Caste:

Education level:

Job:

Section 2: Semi-structured interview

1) This line represents your life from the point you were born (on the left) to now (the right). Please comment on the major changes (improvements or otherwise) to the health of people in your village that have taken place over the course of your life. Please explain why these measures were beneficial.

Today

Your birth

2) How would you describe the health of your village and local area at present?

3) Who do you and your family see for your health needs? What kind of services/treatments do you see them for and why?

3) Have you heard about the Village Alive Project and the Rural Health Facilitators in neighboring villages?

- What have you heard?

4) What have been your and your village’s impressions of the village alive project and the Rural Health Facilitators?

- - Why is this?

5) What are your impressions on the difference in roles between the government community health program and the VAP Rural Health Facilitators?

- Do you believe the two workers complement each other’s work?
- Do you have any examples of this?

6) What are the biggest health challenges that the area currently faces?

- Do you think the Village alive project has the ability to address these challenges?
  - If so, why and how?
  - If not, why not?

7) What other challenges are being faced by the village at present?

- Any discrimination?
- Do you think the Village alive project has the ability to address these challenges?
  - If so, why and how?
  - If not, why not?

8) Do you have any concerns about the Village Alive Project coming to your village?

- If so, what and why?
