# Supplementary material for: Assessing the health impacts of implementing a ‘Comprehensive Rural Health Project’ health system in a low-income region of rural Nepal
Source: PLOS Glob Public Health. 2025 Apr 29;5(4):e0004458. doi: 10.1371/journal.pgph.0004458 (PMC12040125; doi:10.1371/journal.pgph.0004458)
Supplement: S1 Table — This questionnaire was part of a separate quantitative study which was not carried further primary due to unreliable overall data quality. Some issues with the quality of the responses were noted by FJB upon observing the transcribed data. Unfortunately, the interviewer RJ was not aware of the requirement to always request a specific response from the participant. In such cases, the given answer had to be inferred by FB from the overall interview (marked in the table with a ‘?’). Secondly, certain responses such as ‘health center’ or ‘hospital’ were ambiguous, as they appeared to be used interchangeably by participants in interviews during out our qualitative paper. No further analyses were carried out on these findings as a result; they have been included here for the sake of transparency. (DOCX) [file pgph.0004458.s004.docx]

**S1 Table - Supplementary table 1**

[Legend] The table shows results from a brief questionnaire of all included participants. This questionnaire was part of a separate quantitative study which was not carried further primary due to unreliable overall data quality. Some issues with the quality of the responses were noted by FJB upon observing the transcribed data. Unfortunately, the interviewer RJ was not aware of the requirement to always request a specific response from the participant. In such cases, the given answer had to be inferred by FB from the overall interview (marked in the table with a ‘?’). Secondly, certain responses such as ‘health center’ or ‘hospital’ were ambiguous, as they appeared to be used interchangeably by participants in interviews during out our qualitative paper. No further analyses were carried out on these findings as a result; they have been included here for the sake of transparency.

| Participant | Q1: “How has the health of the population changed since the start of the Village Alive Project?” | Q2: “How has access to healthcare changed since the start of the Village Alive Project?” | Q3 “If you get sick who do you normally visit to try to get better?” | Q3 “If your children get sick who do you normally visit to try to get better?” | Q5 Who would you see (if any) for antenatal care if you or your wife got pregnant? |
| --- | --- | --- | --- | --- | --- |
| RHF1 | ?Improved | N/A | Doctor | Doctor | Hospital |
| RHF2 | Much improved | Much improved | Hospital | Hospital | Midwife |
| RHF3 | Much improved | Much improved | Hospital | Hospital | Birthing center |
| RHF4 | ?Improved | Improved | Hospital | Hospital | Hospital |
| RHF5 | Much improved | ?Improved | Primary health post | Hospital | Hospital |
| RHF6 | Much improved | Much improved | Hospital | Hospital | Hospital |
| RHF7 | Much improved | Much improved | Hospital | Hospital | Hospital |
| RHF8 | Much improved | Much improved | Hospital | Hospital | “Home” |
| RHF9 | N/A | ?Improved | Hospital | Hospital | Hospital |
| RHF10 | Much improved | N/A | N/A | Private doctor | Hospital |
| RHF11 | Much improved | N/A | N/A | N/A | Health post |
| RHF12 | Much improved | Much improved | Traditional healer | Private doctor | Hospital |
| Villager 1 | Much improved | Much improved | “Medical shop” | Hospital | Hospital |
| Villager 2 | Much improved | Much improved | Doctor | Hospital | Hospital |
| Villager 3 | Much improved | ?Improved | RHF | Hospital | Hospital |
| Villager 4 | Much improved | Much improved | Hospital | Hospital | Hospital |
| Villager 5 | Much improved | ?Improved | Hospital | Hospital | Hospital |
| Villager 6 | N/A | N/A | N/A | N/A | N/A |
| Villager 7 | N/A | Much improved | Health post | Health post | Hospital |
| Villager 8 | Much improved | Improved | Traditional healer | Hospital | Hospital |
| Villager 9 | N/A | ?Improved | Private doctor | Doctor | Hospital |
| Villager 10 | Much improved | Much improved | Doctor | treatment locally | Hospital |
| Villager 11 | ?Improved | Much improved | N/A | Doctor | Private doctor |
| Villager 12 | Improved | Much improved | RHF | Health post | Hospital |
| Villager 13 | ?Improved | ?Improved | Local doctor | Local doctor | Health post |
| Villager 14 | N/A | ?Improved | private doctor | Private doctor | Health post |
| Villager 15 | Much improved | ?Improved | “Either faith healer or health post” | Hospital | Hospital |
| Villager 16 | Much improved | Much improved | Private doctor | Private doctor | Hospital |
| Villager 17 | Much improved | Much improved | RHF | Hospital | Hospital |
| Villager 18 | ?Improved | Much improved | Private doctor | Private doctor | Hospital |
| Villager 19 | N/A | N/A | N/A | Hospital | Hospital |
| Villager 20 | Much improved | Much improved | Retailer (?Pharmacist) | Hospital | Hospital |
| Villager 21 | ?Improved | ?Improved | Hospital | Hospital | Hospital |
| Villager 22 | ?Improved | ?Improved | Doctor | Hospital | Hospital |
| Villager 23 | Much improved | Much improved | Doctor | Hospital | Doctor |
| Villager 24 | Much improved | “Improved but more costly” | Hospital | Private doctor | Hospital |
| Control villager 1 | N/A | ?Improved | Hospital | Hospital | Hospital |
| Control villager 2 | Much improved | ?Much improved | Doctor | Doctor | Hospital |
| Control villager 3 | Much improved | N/A | Hospital | Doctor | Hospital |
| Control villager 4 | Much improved | N/A | Treatment locally' | “Treatment locally” | Doctor |
| Control villager 5 | Much improved | ?Improved | “Local medicine shop or hospital” | “Local medicine shop or hospital” | “Home then hospital if no success” |
| Control villager 6 | N/A | Improved | Hospital | Hospital | Hospital |
