# Supplementary material for: Assessing the health impacts of implementing a ‘Comprehensive Rural Health Project’ health system in a low-income region of rural Nepal
Source: PLOS Glob Public Health. 2025 Apr 29;5(4):e0004458. doi: 10.1371/journal.pgph.0004458 (PMC12040125; doi:10.1371/journal.pgph.0004458)
Supplement: S2 Table — The middle row reflects the number of transcript files (interviews) with this particular code. The right row is the number of times the code was coded overall. (DOCX) [file pgph.0004458.s005.docx]

**S2 Table - Supplementary table 2**

[Legend] List of initial transcript codes from across VAP villages. The middle row reflects the number of transcript files (interviews) with this particular code. The right row is the number of times the code was coded overall.

| Code | Files with this code | Total code count |
| --- | --- | --- |
| \| Improving social or mental wellbeing \|  \|  \|  \| \| --- \| --- \| --- \| --- \| | 6 | 6 |
| \| Access to healthcare generally \|  \|  \| \| --- \| --- \| --- \| | 1 | 2 |
| \| Accessibility of RHFs vs government programs \|  \|  \| \| --- \| --- \| --- \| | 26 | 41 |
| \| Availability of medicines public vs private \|  \|  \| \| --- \| --- \| --- \| | 1 | 1 |
| \| Benefits of having a stake in the community \|  \|  \| \| --- \| --- \| --- \| | 6 | 7 |
| \| Cost of private healthcare \|  \|  \| \| --- \| --- \| --- \| | 3 | 3 |
| \| Helping with dispensing medication \|  \|  \| \| --- \| --- \| --- \| | 1 | 2 |
| \| Dissatisfaction with government services \|  \|  \| \| --- \| --- \| --- \| | 2 | 2 |
| \| Distrust in state health services \|  \|  \| \| --- \| --- \| --- \| | 9 | 11 |
| \| Increased awareness of available services \|  \|  \| \| --- \| --- \| --- \| | 1 | 2 |
| \| Increased utilization of available services \|  \|  \| \| --- \| --- \| --- \| | 1 | 1 |
| \| Less resistance to accessing services \|  \|  \| \| --- \| --- \| --- \| | 3 | 5 |
| \| Supporting safe childbirth \|  \|  \| \| --- \| --- \| --- \| | 5 | 11 |
|  |  |  |
| \| Cultural changes following arrival of the VAP \|  \|  \| \| --- \| --- \| --- \| | 3 | 4 |
| \| Describing education referring to health \|  \|  \| \| --- \| --- \| --- \| | 1 | 1 |
| \| Having fewer children with better prospects \|  \|  \| \| --- \| --- \| --- \| | 1 | 1 |
| \| Importance of school education \|  \|  \| \| --- \| --- \| --- \| | 7 | 8 |
| \| Non-monetary value of human life \|  \|  \| \| --- \| --- \| --- \| | 2 | 2 |
|  |  |  |
| \| VAP being a point of contact for addressing new challenges \|  \|  \| \| --- \| --- \| --- \| | 2 | 2 |
| \| Addressing inequalities \|  \|  \| \| --- \| --- \| --- \| | 13 | 20 |
| \| Empowering people of lower castes \|  \|  \| \| --- \| --- \| --- \| | 6 | 7 |
| \| Focus on equality for all \|  \|  \| \| --- \| --- \| --- \| | 1 | 1 |
| \| Reducing stigma related to socioeconomic status \|  \|  \| \| --- \| --- \| --- \| | 13 | 17 |
| \| Financial matters \|  \|  \| \| --- \| --- \| --- \| | 17 | 22 |
| \| Roles of the RHF \|  \|  \| \| --- \| --- \| --- \| | 1 | 1 |
| \| Access to sanitation \|  \|  \| \| --- \| --- \| --- \| | 20 | 27 |
| \| Facilities built for healthy living \|  \|  \| \| --- \| --- \| --- \| | 1 | 1 |
| \| Increased knowledge on sanitation \|  \|  \| \| --- \| --- \| --- \| | 24 | 36 |
|  |  |  |
| \| Focus on prevention of illness \|  \|  \| \| --- \| --- \| --- \| | 3 | 3 |
| \| Prevalence of disease in the past \|  \|  \| \| --- \| --- \| --- \| | 13 | 18 |
|  |  |  |
| \| Encouraging engagement with medical services \|  \|  \| \| --- \| --- \| --- \| | 15 | 24 |
| \| Improved identification of diseases \|  \|  \| \| --- \| --- \| --- \| | 1 | 1 |
| \| Facilitating early assessments \|  \|  \| \| --- \| --- \| --- \| | 14 | 19 |
| \| Increased understanding of health \|  \|  \| \| --- \| --- \| --- \| | 18 | 33 |
| \| Lack of healthcare infrastructure in the past \|  \|  \| \| --- \| --- \| --- \| | 4 | 4 |
| \| Providing a source of knowledge for health \|  \|  \| \| --- \| --- \| --- \| | 11 | 14 |
| \| Reducing stigma related to disease \|  \|  \| \| --- \| --- \| --- \| | 6 | 8 |
| \| Traditional vs western medicine \|  \|  \| \| --- \| --- \| --- \| | 17 | 25 |
|  |  |  |
| \| Being fine without RHFs \|  \|  \| \| --- \| --- \| --- \| | 3 | 3 |
| \| Overlap between government and VAP roles \|  \| \| --- \| --- \| | 4 | 4 |
| \| Issues with awareness of RHF's role \|  \|  \| \| --- \| --- \| --- \| | 3 | 4 |
| \| Discrimination against women \|  \|  \| \| --- \| --- \| --- \| | 9 | 10 |
| \| Effectiveness of state health services \|  \|  \| \| --- \| --- \| --- \| | 2 | 2 |
| \| Health affecting financial stability \|  \|  \| \| --- \| --- \| --- \| | 9 | 10 |
| \| Interaction with community leaders \|  \|  \| \| --- \| --- \| --- \| | 1 | 1 |
| \| Discrimination in village \|  \|  \| \| --- \| --- \| --- \| | 1 | 1 |
| \| Positive experiences with women's groups \|  \|  \| \| --- \| --- \| --- \| | 1 | 1 |
| \| Rise of non-communicable diseases \|  \|  \| \| --- \| --- \| --- \| | 1 | 1 |
| \| Suggestions for improving VAP \|  \|  \| \| --- \| --- \| --- \| | 3 | 3 |
| \| Views of other communities \|  \|  \| \| --- \| --- \| --- \| | 1 | 1 |
| \| Health-related ongoing problems \|  \|  \| \| --- \| --- \| --- \| | 10 | 10 |
| \| Issues with providing sanitation \|  \|  \| \| --- \| --- \| --- \| | 3 | 3 |
| \| Issues with running women's groups \|  \|  \| \| --- \| --- \| --- \| | 3 | 3 |
| \| Missed opportunities regarding cooperation \|  \|  \| \| --- \| --- \| --- \| | 1 | 1 |
| \| Non-health ongoing problems \|  \| \| --- \| --- \| | 4 | 5 |
| \| Ongoing stigma (socioeconomic) \|  \|  \| \| --- \| --- \| --- \| | 2 | 2 |
| \| The need for more education \|  \|  \| \| --- \| --- \| --- \| | 4 | 4 |
| \| Issues with money lending program \|  \|  \| \| --- \| --- \| --- \| | 4 | 6 |
| \| Cooperation of RHF and state to solve problems \|  \|  \| \| --- \| --- \| --- \| | 17 | 22 |
| \| Distrust in state health services \|  \|  \| \| --- \| --- \| --- \| | 4 | 4 |
| \| Eagerness to increase responsibility of RHFs \|  \|  \| \| --- \| --- \| --- \| | 8 | 8 |
| \| Facilitating triage \|  \|  \| \| --- \| --- \| --- \| | 5 | 7 |
| \| Holding government services accountable \|  \|  \| \| --- \| --- \| --- \| | 3 | 4 |
| \| Less strain on other health services \|  \|  \| \| --- \| --- \| --- \| | 1 | 1 |
| \| Standing in for unavailable government services \|  \|  \| \| --- \| --- \| --- \| | 2 | 2 |
