# Supplementary material for: Assessing the health impacts of implementing a ‘Comprehensive Rural Health Project’ health system in a low-income region of rural Nepal
Source: PLOS Glob Public Health. 2025 Apr 29;5(4):e0004458. doi: 10.1371/journal.pgph.0004458 (PMC12040125; doi:10.1371/journal.pgph.0004458)
Supplement: S3 Table — The middle column reflects the number of transcript files (interviews) with this particular code. The right column is the number of times the code was coded overall. (DOCX) [file pgph.0004458.s006.docx]

**S3 Table - Supplementary table 3**

[Legend] List of initial transcript codes from across control villages. The middle column reflects the number of transcript files (interviews) with this particular code. The right column is the number of times the code was coded overall.

| Code | Files with this code | Total code count |
| --- | --- | --- |
| \| Prevalence of disease in the past \|  \|  \|  \| \| --- \| --- \| --- \| --- \| | 5 | 8 |
| Current access to state healthcare services | 3 | 6 |
| Financial security improving ability to pay for health | 1 | 3 |
| overall accessibility of treatment vs the past | 2 | 2 |
| Satisfaction with health provision | 1 | 2 |
| Improved knowledge on sanitation | 4 | 4 |
| Improvements in understanding of health | 0 | 1 |
| Acknowledging the importance of early checkups | 2 | 2 |
| Transition from communicable diseases to NCDs | 1 | 1 |
| Change in access to sanitation | 4 | 6 |
| Quality of state healthcare services | 1 | 1 |
| Lack of knowledge on services available | 1 | 1 |
| Stigma related to disease | 1 | 1 |
| Traditional medicine vs evidence based medicine | 6 | 8 |
| Good accessibility of state health staff | 1 | 1 |
| Health affecting financial security | 1 | 1 |
| Lack of control over state health access | 1 | 1 |
| Lack of financial security, relying on others for money | 3 | 4 |
| lack of trust in government health practice | 1 | 1 |
| Satisfaction with health system | 1 | 1 |
| Lack of access to clean water | 1 | 2 |
| Nobody responsible for sanitation | 1 | 2 |
| Ongoing lack of sanitation | 2 | 3 |
| Sanitation infrastructure no longer functioning | 1 | 1 |
| Improved social relations with other villagers | 1 | 1 |
| Lack of discrimination | 1 | 2 |
| Poverty, reliance on destroying land | 2 | 2 |
| Reliance on foreign economies for income | 1 | 1 |
| Aspirations for VAP (non-health) | 2 | 2 |
| Aspired roles of RLH education | 1 | 1 |
| Aspired roles of RLH (medical) | 2 | 2 |
| Eagerness for further health provision | 1 | 1 |
| Lack of person fighting for their community | 1 | 1 |
